# The prevalence and 6-year incidence of myopic tessellation in a Chinese rural adult population: the Handan Eye Study

**DOI:** 10.1186/s12886-025-03874-7

**Published:** 2025-01-31

**Authors:** Caixia Lin, Jian Wu, Kai Cao, Bingsong Wang, Yuxin Fang, Ohno-Matsui Kyoko, Yunyun Sun, Jie Hao, Lixia Ma, Ye Zhang, Qing Zhang, Ningli Wang, the Handan Eye Study Group

**Affiliations:** 1 Beijing Tongren Eye Center, Beijing Tongren Hospital, Beijing Key Laboratory of Ophthalmology and Visual Sciences, Capital Medical University, No. 1 Dong Jiao Min Xiang Street, Beijing, Dongcheng District 100730 P.R. China; 2 Henan Academy of Innovations in Medical Science, Henan, 450008 China; 3 Beijing Institute of Ophthalmology, Beijing Tongren Eye Center, Beijing Tongren Hospital, Capital Medical University, Beijing Key Laboratory of Ophthalmology and Visual Sciences, Beijing, 100730 China; 4 Department of Ophthalmology and Visual Science, Tokyo Medical and Dental University, Tokyo, Japan; 5 The First Affiliated Hospital (College of Clinical Medical) of Henan University of Science and Technology, Luoyang, 471000 China

**Keywords:** Fundus tessellation, Prevalence, Incidence, Risk factors, Longitudinal study

## Abstract

**Purpose:**

To describe the prevalence and the cumulative 6-year incidence of fundus tessellation in a rural Chinese adult population.

**Methods:**

The Handan Eye Study was a population-based longitudinal study, with 6830 eligible subjects included in the baseline period, and 5394 subjects (follow-up rate: 85.3%) took part in the follow-up study. Participants had detailed eye examinations and physical examinations; a detailed questionnaire was also well administered. Fundus tessellation was defined as well-defined choroidal vessels that can be observed clearly around the fovea as well as around the arcade vessels.

**Results:**

Among 6830 subjects who participated in the baseline study, the prevalence of fundus tessellation was 9.89% (95%CI: 9.22-10.58%). The prevalence of fundus tessellation increased with age; that of subjects aged younger than 50 years and those aged 50 years or older was 2.5% and 14.5%, respectively. Six years later, the cumulative 6-year incidence of fundus tessellation was 1.21% (95%CI: 1.03-1.40%). Similarly, the incidence of fundus tessellation also increased with age; that of participants aged younger than 50 years and those aged 50 years or older was 0.20% and 1.86%, respectively. The progression rate of fundus tessellation in those with fundus tessellation at baseline was 1.5% (95% CI: 0.82%, 2.38%). By multivariable analysis, subjects being older (OR: 1.079, *P* < 0.001) and havingβ-parapapillary atrophy at baseline (OR: 2.657, *P* = 0.002) were associated with a higher risk of incidence of fundus tessellation.

**Conclusions:**

The prevalence and 6-year incidence of fundus tessellation were 9.89% and 1.21% in rural Chinese adults aged 30 + years, respectively. The progression rate in participants with fundus tessellation indicates the importance of regular follow-up for these patients.

## Introduction

Fundus tessellation is characterized by the visibility of the underlying choroid vessels around the fovea and arcade vessels. It is considered that axial elongation of the eyeball results in a thinning of the retinal pigment epithelium (RPE) and choriocapillaris, which causes the choroidal vessels to be visible [1]. Previous studies have shown that fundus tessellation is significantly correlated with myopia and myopic retinopathy [2–4]. Victor TC KOH et al. [3] found that 85.7% of young Singaporean men with high myopia (myopia diopter < −10.0D) had fundus tessellation. In 2015, an international photographic classification and grading system for myopic maculopathy proposed by Ohno Matsui et al. defined fundus tessellation as category 1 myopic maculopathy [1]. Although previous studies have shown that myopes with fundus tessellation have better best corrected visual acuity than those with other myopic macular diseases, However, Kengo Hayashi et al. [5] followed up 327 patients (806 eyes) with high myopia for 12.7 years and found that 13.4% of patients (276 eyes) having high myopia (refractive error more than − 8.00D or axial length (AL) ≥ 26.5 mm) with fundus tessellation at baseline had pathological fundus changes (grade II and above myopic maculopathy: diffuse chorioretinal atrophy, patchy chorioretinal atrophy, macular atrophy, lacquer cracks, myopic CNV and Fuchs spot). The above research showed that fundus tessellation has the potential risk of visual impairment.

The Beijing Eye Study [6] in 2011 demonstrated that the mean degree of fundus tessellation in the macular region and peripapillary fundus tessellation of adults 50 years of age or older were 0.84 and 1.01 (the classification of fundus tessellation was 0 to 3). In addition, the research also found that a higher degree of fundus tessellation was associated with older age, male sex, lower body mass index (BMI), thinner sub-foveal choroidal thickness, longer AL, a larger parapapillary beta zone, and a lower prevalence of AMD. Hiroto Terasaki et al. [7] found that the AL in the eyes with infra-temporal fundus tessellation were longer than those without fundus tessellation, and the differences were significant. However, these studies were all cross-sectional studies and couldn’t demonstrate the causal relationship between fundus tessellation and the associated risk factors.

Therefore, this study aims to describe the prevalence, the cumulative 6-year incidence of fundus tessellation, and its associated factors in a rural Chinese adult population to identify the risk factors of grade 1 myopic maculopathy and to intervene the variable risk factors to reduce the progression of fundus tessellation to higher grade myopic maculopathy.

## Methods

The Handan Eye Study was a population-based longitudinal epidemiologic study of eye diseases conducted in Handan, Hebei province, northern China. In 2006–2007, 6830 people aged 30 years or older participated in the baseline study, which had been described in detail elsewhere [8, 9]. In 2012–2013, participants still surviving were invited to take part in a follow-up study, which had been described in detail too [10]. The study protocol was approved by the Beijing Tongren Hospital Ethics Committee (TREC2006-22), and written informed consent was obtained from all the participants, according to the Declaration of Helsinki.

In both the baseline and follow-up studies, the participants experienced a detailed eye examination that included visual acuity (VA), intraocular pressure (IOP), fundus photographs, and so on, and a physical examination including height and weight according to a similar protocol.

### Eye examination and interviews

Presenting VA (i.e., wearing habitual correction, if any) was measured binocularly, then monocularly (right eye followed by left eye) according to the Early Treatment Diabetic Retinopathy testing protocol with a log MAR chart under standardized lighting conditions at 4 m. For subjects who could not see the chart at 4 m, VA was tested at 1 m, allowing acuities as low as 1/40 (0.025) to be tested. If no letters were identified on the chart, VA was assessed for the ability to count fingers, see hand movements, or perceive light. Subjective refraction was performed on all the participants whose VA was worse than 0.0 log MAR (Snellen 6/6 or 20/20) by trained and certified study optometrists. Autorefractor (KR8800; Topcon, www.global.topcon.com) readings were used as the starting point, and reconfiguration of sphere, cylinder, and axis was performed until the best corrected VA (BCVA) was obtained.

Intraocular pressure (IOP) was measured using the Kowa applanation tonometer HA-2 (Kowa Company Ltd., Tokyo, Japan) in cooperative people. Those who couldn’t cooperate were measured by a Schiotz tonometer or digital palpation [11]. AL, anterior chamber depth (ACD), and lens thickness (LT) were measured using a 10 MHz A/B-mode ultrasound device (Cine Scan, www.quantel-medical.com). The degree of lenticular opacity was graded by study ophthalmologists according to LOCS III with slit lamp microscopy (Topcon SL-2 F; www.global.topc.com) after pupil dilation.

At baseline, dilated 45° digital color fundus photographs of Early Treatment Diabetic Retinopathy standard field 1 [12] (centered on the optic disc, stereoscopic) and standard field 2 (centered on the macula, non-stereoscopic) were taken for each eye by trained and certified photographers. One-third of the fundus photographs were taken using a Topcon TRC-NW6S/7S (Topcon, www.global.topcon.com) camera (during the initial stages of the baseline study). And the other two-thirds were taken using a Canon CRDGi with a 20D SLR back (Canon, www.canon.com). In the follow-up study, a Canon CR 2 with a 20D SLR back (Canon, www.ca-non.com) was used for all the participants.

Height and weight were measured according to the standard operating procedure of a protocol by certified nurses at both baseline and follow-up study [13]. In addition, a detailed, interviewer-administered questionnaire including demographic information (educational level, marital status, last year’s personal income, and so on), medical conditions (history of diabetes, hypertension, drinking, and smoking), as well as family history of eye diseases, was used.

### Definitions

Myopia was defined as spherical equivalent (SE, sphere + 1⁄2 cylinder) less than − 0.5 diopters (D), and high myopia as SE less than − 5.0 D. Change SE was defined as SE at follow-up study minus SE at baseline study. Fundus tessellation related to myopia was defined as having well-defined choroidal vessels that can be observed clearly around the fovea as well as the arcade vessels [1]. Parapapillary atrophy was evaluated by the method mentioned by Jonas et al. [14]. Diabetes and hypertension were defined according to self-reports from participants (previously diagnosed). Educational level was divided into seven levels: illiteracy, half-illiteracy (< 1year), primary school (1–5 years), middle school (6–8 years), high school (9–11 years), mid-technical school, and college. BMI is equal to weight divided by height squared.

**Fig. 1:**
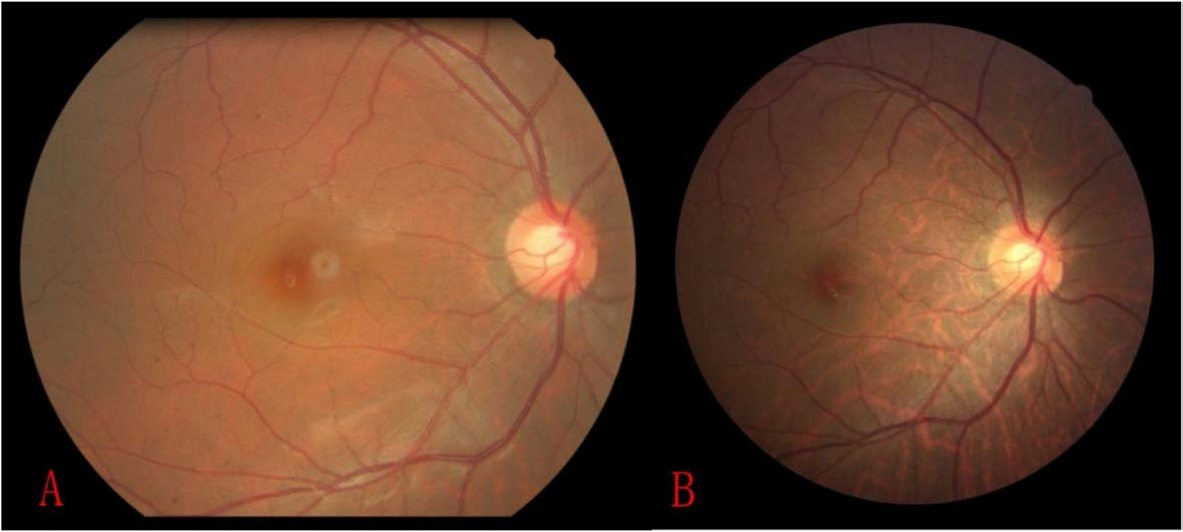
Comparative follow-up of fundus photographs. A: The fundus photographs of baseline without fundus tessellation. B: The subject developed fundus tessellation in the right eye after 6 years of follow-up

If a participant had at least one eye with fundus tessellation, then he was regarded as having fundus tessellation. A new-onset fundus tessellation was defined as the absence of fundus tessellation in both eyes of a participant at baseline while the presence of fundus tessellation in at least one eye of him/her at follow-up 6 years later (Fig. 1. A and B).

### Fundus photography grading

Fundus photographs at baseline and follow-up study were graded by a grader (C.X.L) who had been trained and certified for myopic maculopathy grading by a retinal specialist (K.O.M.) [15] For the photographs with fundus tessellation, the grader discussed with the other two graders (B.S.W and Y.X.F., trained and certified) and conformed the results. If there were still different opinions after discussion, a retinal specialist (K.O.M) reassessed the relevant photographs and made a final decision. Lastly, the specialist checked all the fundus photographs classified as fundus tessellation to confirm the final grading result. All graders were masked to information such as the participants’ refractive error and AL to minimize bias. Initially, photographs from the baseline and follow-up study were graded separately. Subsequently, for photographs diagnosed inconsistently in fundus tessellation between baseline and follow-up, side-by-side comparisons were made between these photographs; during the process, graders were masked to whether the photographs were taken at baseline or follow-up [16]. To determine intra-grader variability, the grader (C.X.L) randomly selected the images of 200 eyes of 100 participants and graded them twice in a masked manner with an interval of 2 weeks.

### Statistics

Statistical analyses were performed using SAS software (version 9.1.4, SAS Institute Inc.; Cary, North Carolina). Statistical analyses included the Wilcoxon test, χ2 test, and logistic regression. The prevalence and incidence of fundus tessellation were analyzed by person (person-specific), and the corresponding 95% confidence intervals (CIs) were reported. A binary logistic regression model was used to analyze the risk factors of new-onset fundus tessellation. First, univariate correlation analyses were carried out to identify ocular and systemic parameters associated with new-onset fundus tessellation. Then, multivariate logistic regression analysis was performed, with new-onset fundus tessellation as the dependent variable and the parameter with *P* < 0.1 in univariate analysis as the independent variable. If the participant had fundus tessellation in both eyes, the generalized estimation model was used to correct. The SMOTE model was used to correct the unbalanced dependent variable parameter [17–19]. The intra-grader weighted kappa value was 0.78 for the assessment of fundus tessellation.

## Results

### Baseline prevalence of fundus tessellation

**Fig. 2:**
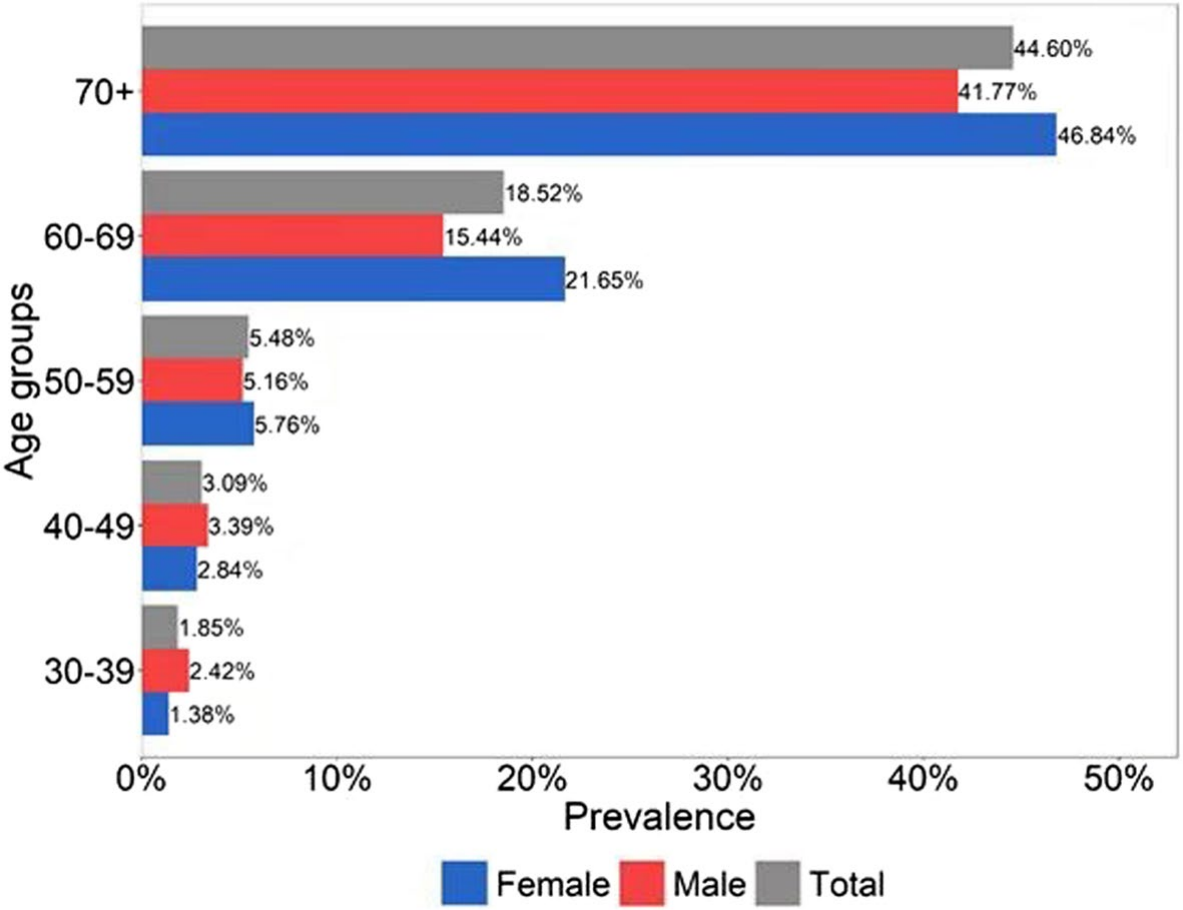
The incidence rates of fundus tessellation between men and women across different age groups

Of the 6,830 people who participated in the Handan Eye Baseline Study, 6453 people had at least one clear fundus photograph to grade after those having no photographs or refractive interstitial opacity that caused the photograph couldn’t to be graded were removed. Among them, 638 subjects (1042 eye) had fundus tessellation, and the total prevalence of the population was 9.89% (95%CI: 9.22 − 10.58%). The age-standardized prevalence was 8.93% in males and 10.64% in females. The total age-sex standardized prevalence was 9.77%. The prevalence of the male population was 9.16% (95%CI: 8.23 − 10.14%), and that of the female was 10.52% (95%CI: 9.58 − 11.50%). The prevalence of women was higher than that of men, but there was no significant difference between them (*P* = 0.069). The prevalence of fundus tessellation in different age groups of men, women, and the total population was presented in Table 1. From Table 1; Fig. 2, we can see that before 50 years old, the prevalence of fundus tessellation in men was higher than that of women, while after 50 years old, the prevalence of fundus tessellation in women was higher than that of men. And after 60 years old the prevalence of fundus tessellation in both men and women increased markedly. The prevalence was 2.5% in people under 50 years old, and 14.5% in people over 50 years old. The prevalence of fundus tessellation in people over 50 years old was significantly higher than that in people under 50 years old. There was a significant statistical difference between them (*P* < 0.001).

**Table 1:** Prevalence of fundus tessellation in male, female and both at Handan Eye Baseline Study

Gender	Age group	*N*	Fundus tessellation	Prevalence	95%CI	
**Male**	30–39	537	13	2.42%	1.57%	3.45%
	40–49	590	20	3.39%	2.35%	4.62%
	50–59	1105	57	5.16%	4.12%	6.31%
	60–69	531	82	15.44%	12.55%	18.58%
	≥ 70	249	104	41.77%	36.23%	47.41%
	**Total**	**3012**	**276**	**9.16%**	**8.23%**	**10.14%**
**Female**	30–39	650	9	1.38%	0.87%	2.02%
	40–49	703	20	2.84%	2.00%	3.84%
	50–59	1250	72	5.76%	4.70%	6.92%
	60–69	522	113	21.65%	18.22%	25.28%
	≥ 70	316	148	46.84%	42.12%	51.58%
	**Total**	**3441**	**362**	**10.52%**	**9.58%**	**11.50%**
**Male + Female**	30–39	1187	22	1.85%	1.36%	2.42%
	40–49	1293	40	3.09%	2.41%	3.85%
	50–59	2355	129	5.48%	4.72%	6.28%
	60–69	1053	195	18.52%	16.24%	20.91%
	≥ 70	565	252	44.60%	40.99%	48.25%
	**Total**	**6453**	**638**	**9.89%**	**9.22%**	**10.58%**

### Follow-up participation and comparison

Six years later, of the 6830 people who participated in the Handan Eye baseline study, 507 (7.4%) died. Of the remaining 6323 survivors, 929 were lost to follow-up. Ultimately, 5394 participants (follow-up rate: 85.3%) took part in the Handan Eye Follow-up Study; among them, 5048 participants (9976 eyes) had readable fundus photographs in at least one eye both at baseline and in the follow-up study. Table 2 showed that compared with participants who were lost to follow-up or had no fundus photographs, those who participated were much younger (51.2 vs. 55.6, *P* < 0.001), had more female (55.1% vs. 49.8%, *P* < 0.001), got higher education level (*P* < 0.001), had higher marriage rate (90.5% vs. 84.8%, *P* < 0.001), higher personal income (*P* < 0.001), higher BMI (24.6 vs. 24.3, *P* = 0.038), lower DM prevalence (5.98% vs. 10.2%, *P* < 0.001), higher smoke (27.2% vs. 25.1%, *P* = 0.031) and drink (18.7% vs. 16.4%, *P* = 0.043) prevalence, better PVA OD (0.8 vs. 0.63, *P* < 0.001), higher spherical equivalent refraction (SER) OD (*P* = 0.006), higher β-PPA prevalence (34.1% vs. 31.2%, *P* < 0.001 ) and lower nuclear opacity (NO, *P* < 0.001) at baseline. While these two groups had no significant differences in hypertension prevalence, AL OD at baseline, or change in SER.

**Table 2:** Comparisons of demographic characteristics, ocular and systemic parameters of people included and excluded in Handan Eye follow-up study

Variables	Overall	Included	Excluded	*P*
	(*N* = 6830)	(*N* = 5048)	(*N* = 1782)	
**Age (Years)**				< 0.001
Mean (SD)	52.3 (12.1)	51.2 (10.9)	55.6 (14.6)	
**Gender**				< 0.001
male	3163 (46.3%)	2269 (44.9%)	894 (50.2%)	
female	3667 (53.7%)	2779 (55.1%)	888 (49.8%)	
**Education level**				< 0.001
illiteracy	797 (11.7%)	522 (10.3%)	275 (15.4%)	
semi- illiteracy	300 (4.4%)	195 (3.9%)	105 (5.9%)	
primary	3401 (49.8%)	2578 (51.1%)	823 (46.2%)	
middle school	2129 (31.2%)	1605 (31.8%)	524 (29.4%)	
high school	191 (2.8%)	137 (2.7%)	54 (3.0%)	
mid technique school	10 (0.1%)	9 (0.2%)	1 (0.1%)	
college	2 (0.0%)	2 (0.0%)	0 (0%)	
**Marital status**				< 0.001
single	96 (1.4%)	56 (1.1%)	40 (2.2%)	
married	6078 (89.0%)	4567 (90.5%)	1511 (84.8%)	
divorced	16 (0.2%)	11 (0.2%)	5 (0.3%)	
re-marriage	97 (1.4%)	79 (1.6%)	18 (1.0%)	
widow	543 (8.0%)	335 (6.6%)	208 (11.7%)	
**Last year’s personal income**				< 0.001
no income	1867 (27.3%)	1315 (26.1%)	552 (31.0%)	
<1800	346 (5.1%)	266 (5.3%)	80 (4.5%)	
<2500	468 (6.9%)	390 (7.7%)	78 (4.4%)	
<3500	515 (7.5%)	390 (7.7%)	125 (7.0%)	
<5000	970 (14.2%)	765 (15.2%)	205 (11.5%)	
<9000	544 (8.0%)	419 (8.3%)	125 (7.0%)	
>=9000	670 (9.8%)	484 (9.6%)	186 (10.4%)	
refuse	366 (5.4%)	304 (6.0%)	62 (3.5%)	
not sure	1084 (15.8%)	715 (14.2%)	369 (20.7%)	
**BMI (Kg/m2)**				0.038
Mean (SD)	24.5 (3.77)	24.6 (3.60)	24.3 (4.25)	
**DM**	387 (6.91%)	262 (5.98%)	125 (10.2%)	< 0.001
**Hypertension**	1339 (19.6%)	980 (19.4%)	359 (20.1%)	0.526
**Smoke**	1818 (26.6%)	1371 (27.2%)	447 (25.1%)	0.031
**Drink**	1234 (18.1%)	942 (18.7%)	292 (16.4%)	0.043
**PVA OD**				< 0.001
Median (Q1, Q3)	0.1 (0.3, 0)	0.1 (0.3, 0)	0.2 (0.4, 0)	
**AL OD**				0.334
Median (Q1, Q3)	22.8 (22.3, 23.3)	22.8 (22.3, 23.3)	22.8 (22.3, 23.3)	
**SER OD**				0.006
Median (Q1, Q3)	0.00 (−0.50, 0.50)	0.00 (−0.50, 0.50)	0.00 (−0.625, 0.625)	
**Change in SER (D)**				0.437
Median (Q1, Q3)	0.250 (−0.125,0.625)	0.250 (−0.125,0.625)	0.250 (−0.483,0.750)	
**β_PPA**				< 0.001
NO	4059 (59.4%)	3254 (64.5%)	805 (45.2%)	
YES	2277 (33.3%)	1721 (34.1%)	556 (31.2%)	
Missing	494 (7.2%)	73 (1.4%)	421 (23.6%)	
**NO OD**				< 0.001
Median (Q1, Q3)	2 (1.5, 2.5)	2 (1.5, 2.5)	2 (1.5, 3)	

Continuous data are expressed as Mean ± SD or median (inter-quartile range) according to normality test. Categorical data are expressed as n (%)

*BMI* Body mass index, *DM* Diabetes, *PVA* Present VA, *AL* Axial length, *SER* Spherical equivalent refraction, *β_PPA* means having β_PPA at baseline, *NO* Nuclear opacity

### Incidence of fundus tessellation over six years

Six years later, 61 subjects (81 eyes) had new fundus tessellation (Table 3). The age-standardized incidence of fundus tessellation was 1.27% in males and 1.35% in females. The age-sex standardized incidence was 1.32%. The total incidence of fundus tessellation in 6 years was 1.21% (95% CI: 1.03 − 1.40%). The incidence of fundus tessellation in men and women was 1.15% (95% CI: 0.89 − 1.43%) and 1.26% (95% CI: 1.01 − 1.53%), respectively. And there was no significant difference in the incidence of fundus tessellation between males and females (*P* = 0.713). In 30–39 and 60–69 age groups, the incidence of fundus tessellation in males was higher than that in the females, and in 40–49, 50–59, and ≥ 70 age groups, the incidence of fundus tessellation in females was higher than that in the male. The difference in the incidence of fundus tessellation in the 50–59 age group was significant between males and females (*P* = 0.038), while in other age groups there were no significant difference between males and females (*P* = 0.101, *P* = 0.507, *P* = 0.165, and *P* = 0.682, respectively). The incidence of fundus tessellation was 0.20% in people younger than 50 years old and 1.86% in people older than 50 years old. And the incidence of fundus tessellation in people aged 50 years and above was significantly higher than that in people under 50 years old (*P* < 0.001).

**Table 3:** Incidence of fundus tessellation at Handan Eye follow-up study

Gender	Age group	*N*	New fundus tessellation	Incidence	95%CI	
**Male**	30–39	389	2	0.51%	0.21%	0.95%
	40–49	485	0	0.00%	0.00%	0.00%
	50–59	884	4	0.45%	0.26%	0.70%
	60–69	391	14	3.58%	2.28%	5.17%
	≥ 70	120	6	5.00%	2.26%	8.75%
	**Total**	2269	26	1.15%	0.89%	1.43%
**Female**	30–39	504	0	0.00%	0.00%	0.00%
	40–49	613	2	0.33%	0.15%	0.57%
	50–59	1088	15	1.38%	0.97%	1.86%
	60–69	413	8	1.94%	1.12%	2.97%
	≥ 70	161	10	6.21%	3.38%	9.83%
	**Total**	2779	35	1.26%	1.01%	1.53%
**Male + Female**	30–39	893	2	0.22%	0.11%	0.37%
	40–49	1098	2	0.18%	0.09%	0.30%
	50–59	1972	19	0.96%	0.72%	1.24%
	60–69	804	22	2.74%	1.96%	3.64%
	≥ 70	281	16	5.69%	3.60%	8.23%
	**Total**	**5048**	**61**	**1.21%**	**1.03%**	**1.40%**

#### Progression from fundus tessellation to myopic maculopathy

Of 638 people (1042 eyes) with fundus tessellation at baseline, 400 (647 eyes) participated in the follow-up study 6 years later, and 6 (8 eyes) developed myopic maculopathy (diffuse chorioretinal atrophy), with a 6-year progression rate of 1.5% (95%CI: 0.82%, 2.38%) for fundus tessellation. Compared to those without new-onset fundus tessellation(Table 4), participants with new-onset were much older (61.8 vs. 51.0, *P* < 0.001), had lower educational level (*P* = 0.004), lower marriage rate (80.3% vs. 90.6%, *P* = 0.048), lower last year’s personal income (*P* < 0.001), higher DM (12.7% vs. 5.9%, *P* = 0.044) and hypertension (31.1% vs. 19.3%, *P* = 0.030) prevalence, worse PVA OD (0.5 vs. 0.8, *P* < 0.001), higherβ-PPA prevalence (59.0% vs. 33.8%, *P* < 0.001), and higher NO OD (2.5 vs. 2, *P* < 0.001) at baseline study. While there was no significant difference in gender, BMI, smoke and drink prevalence, AL OD, and SER OD at baseline, there was a change in SER between the two groups.

**Table 4:** Comparisons of demographic characteristics, ocular and systemic parameters between participants with or without new-onset fundus tessellation at Handan Eye follow-up study

Variables	Overall	New fundus tessellation	No fundus tessellation	*P*
	(*N* = 5048)	(*N* = 61)	(*N* = 4987)	
**Age (year)**				< 0.001
Mean (SD)	51.2 (10.9)	61.8 (9.47)	51.0 (10.9)	
**Gender**				0.812
male	2269 (44.9%)	26 (42.6%)	2243 (45.0%)	
female	2779 (55.1%)	35 (57.4%)	2744 (55.0%)	
**Education level**				0.004
illiteracy	522 (10.3%)	15 (24.6%)	507 (10.2%)	
semi- illiteracy	195 (3.9%)	1 (1.6%)	194 (3.9%)	
primary	2578 (51.1%)	34 (55.7%)	2544 (51.0%)	
middle school	1605 (31.8%)	9 (14.8%)	1596 (32.0%)	
high school	137 (2.7%)	2 (3.3%)	135 (2.7%)	
mid technique school	9 (0.2%)	0 (0%)	9 (0.2%)	
college	2 (0.0%)	0 (0%)	2 (0.0%)	
**Marital status**				0.048
single	56 (1.1%)	2 (3.3%)	54 (1.1%)	
married	4567 (90.5%)	49 (80.3%)	4518 (90.6%)	
divorced	11 (0.2%)	0 (0%)	11 (0.2%)	
re-marrige	79 (1.6%)	1 (1.6%)	78 (1.6%)	
widow	335 (6.6%)	9 (14.8%)	326 (6.5%)	
**Last year’s personal income**				< 0.001
no income	1315 (26.1%)	32 (52.5%)	1283 (25.7%)	
<1800	266 (5.3%)	5 (8.2%)	261 (5.2%)	
<2500	390 (7.7%)	1 (1.6%)	389 (7.8%)	
<3500	390 (7.7%)	3 (4.9%)	387 (7.8%)	
<5000	765 (15.2%)	6 (9.8%)	759 (15.2%)	
<9000	419 (8.3%)	3 (4.9%)	416 (8.3%)	
>=9000	484 (9.6%)	1 (1.6%)	483 (9.7%)	
refuse	304 (6.0%)	6 (9.8%)	298 (6.0%)	
not sure	715 (14.2%)	4 (6.6%)	711 (14.3%)	
**BMI (Kg/m**^**2**^**)**				0.964
Mean (SD)	24.6 (3.60)	24.6 (3.89)	24.6 (3.59)	
**DM**	262 (5.98%)	7 (12.7%)	255 (5.90%)	0.044
**Hypertension**	980 (19.4%)	19 (31.1%)	961 (19.3%)	0.030
**Smoke**	1371 (27.2%)	13 (21.3%)	1358 (27.2%)	0.130
**Drink**	942 (18.7%)	5 (8.20%)	937 (18.8%)	0.077
**PVA OD**				< 0.001
Median (Q1, Q3)	0.1 (0.3,0)	0.3 (0.5,0.1)	0.1 (0.3,0)	
**AL OD**				0.245
Median (Q1, Q3)	22.8 (22.3, 23.3)	22.6 (22.3, 23.0)	22.8 (22.3, 23.3)	
**SER OD**				0.287
Median (Q1, Q3)	0.00 (−0.50, 0.50)	0.25 (−0.75, 0.91)	0.00 (−0.50, 0.50)	
**Change in SER (D)**				0.362
Median (Q1, Q3)	0.250 (−0.125,0.625)	−0.125 (−0.590,0.375)	0.250 (−0.125,0.625)	
**β_PPA**				< 0.001
NO	3254 (64.5%)	18 (29.5%)	3236 (64.9%)	
YES	1721 (34.1%)	36 (59.0%)	1685 (33.8%)	
Missing	73 (1.4%)	7 (11.5%)	66 (1.3%)	
**NO OD**				< 0.001
Median (Q1, Q3)	2 (1.5,2.5)	2.5 (2,3)	2 (1.5,2.5)	

Continuous data are expressed as Mean ± SD or median (inter-quartile range) according to normality test. Categorical data are expressed as n (%)

#### Risk factors for new-onset fundus tessellation

**Fig. 3:**
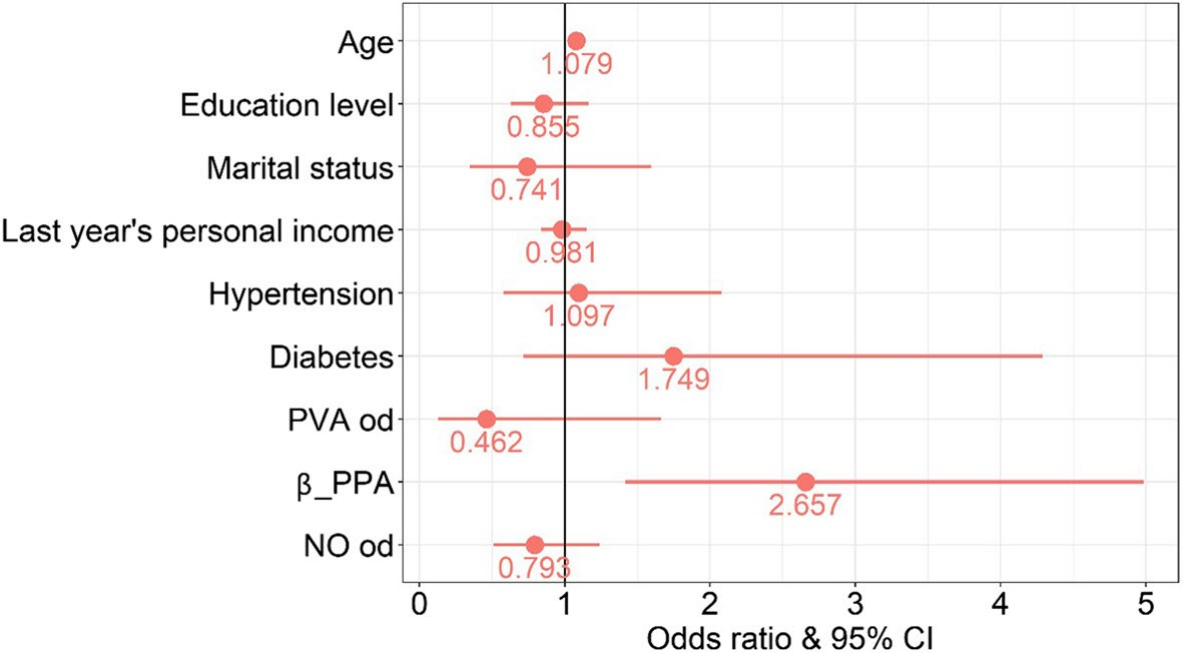
The forest map of the risk factors in fundus tessellation

In multivariate logistic regression analysis, the new-onset fundus tessellation was taken as the dependent variable, and the related factors with *P* < 0.1 in the univariate analysis were taken as the independent variables. The results showed that there were still significant correlations between new-onset fundus tessellation and advanced age (*P* < 0.001), baseline β-PPA (*P* = 0.002) (Table 5). Participants with baseline β-PPA (OR: 2.657, 95%CI: 1.417, 4.982), and older age (OR: 1.079, 95%CI: 1.034, 1.127) were more likely to have new-onset fundus tessellation than participants without these characteristics. The logistic regression forest map (Fig. 3) presented the result intuitively.

**Table 5:** Logistic regression analysis of risk factors of new on-set fundus tessellation

Variables	OR	95%CI		*P*
**Age**	1.079	1.034	1.127	< 0.001
**Education level**	0.855	0.628	1.164	0.320
**Marital status**	0.741	0.345	1.592	0.442
**Last year’s personal income**	0.981	0.836	1.151	0.816
**Hypertension**	1.097	0.579	2.078	0.776
**Diabetes**	1.749	0.713	4.290	0.222
**PVA OD**	0.462	0.128	1.666	0.238
**β_PPA**	2.657	1.417	4.982	0.002
**NO OD**	0.793	0.509	1.237	0.307

## Discussion

Our study described the prevalence and incidence of fundus tessellation in a population-based Chinese adult population. The results showed that the prevalence of fundus tessellation was 9.89% and the 6-year incidence was 1.21% in a Chinese rural adult population. The occurrence of fundus tessellation was related to age and baseline β-PPA. People with older age and β-PPA at baseline were more likely to have new-onset fundus tessellation than others.

Our study showed that the prevalence of fundus tessellation increased significantly with age. The prevalence of fundus tessellation was 2.5% and 14.5% in people aged 30–49 years old and over 50 years old, respectively. Especially after 60-year-old, there was an evident increase in the prevalence of fundus tessellation. Our previous research [20] also showed that the prevalence of myopic retinopathy was correlated with age significantly. And the prevalence of myopic retinopathy in people age 50 and older was 0.4% and 1.2%, respectively. In addition, our study demonstrated that the incidence of fundus tessellation increased with age. The incidence of fundus tessellation in people aged 30–49 years old and over 50 years old was 0.20% and 1.86%, respectively. The prevalence of fundus tessellation in older adults was higher than that of the youngers, which was explained by the incidence of fundus tessellation in different age groups. The main reason for the increasing prevalence of fundus tessellation and myopic retinopathy with age is that pathological myopia is an age-related disease; that is, pathological myopia progresses and worsens with age. Kengo Hayashi et al. [5] found that 40.6% of the patients with highly myopia (327 out of 806 eyes) showed a progression of the myopic maculopathy during a mean follow-up of 12.7 years. Although fundus tessellation does not belong to pathologic myopia, it belongs to the first level of myopic maculopathy, which also has the characteristics of continuous change and progress as time goes on.

The prevalence of fundus tessellation in adults over 30 years old was 9.89% in our study, which was significantly lower than that of young men with high myopia (myopia diopter < −10.0D) in Singapore (85.7%) [3]. The possible reason was that the population they focused on was mainly patients with high myopia. As the AL of patients with high myopia becomes longer, the retina, choroidal capillary layer, and other structures are stretched and thinned, thus revealing the underlying choroidal large blood vessels and showing the changes of fundus tessellation. However, in our study, the proportion of people with myopia was low (prevalence of myopia: 26.7%, average SE: −0.14 (1.75) D), so the prevalence of fundus tessellation was low. Whether the prevalence of fundus tessellation is low in our study compared with other populations needs to be confirmed by further population-based epidemiological studies.

The incidence of fundus tessellation in 6 years in our study was 1.21%. And the incidence of fundus tessellation increased with age, especially after 60 years old. The reason may be that with the increase of age, the tissues and organs of the body decay, and the RPE of the retina and the choroidal capillary layer also degenerate, which leads to the penetration of the choroidal vessels, that is, the appearance of fundus tessellation.

Among those participants who had fundus tessellation at baseline, 1.5% of them developed myopic maculopathy (diffuse chorioretinal atrophy) at follow-up study 6 years later. Kengo Hayashi et al. [5] found that 13.4% of high myopia patients with fundus tessellation developed myopic maculopathy after 12.7 years of follow-up (10.1% progressed to diffuse chorioretinal atrophy, 2.9% to lacquer cracks, and 0.4% to myopic CNV). The progression rate of fundus tessellation was significantly higher than that of our population. The reason may be that the people studied by Kengo Hayashi et al. were highly myopic patients who had a long AL and were more likely to develop myopic maculopathy over time. Besides, Kengo Hayashi et al. [5] followed up the patients for an average of 12.7 years, while our study followed up the population only for 6 years. Previous studies have shown that the progression rate of myopic maculopathy was low [15, 21, 22]. Therefore, long-term follow-up observation is more likely to see the progression of the disease. Besides, further studies are also needed to confirm whether the progression rate of fundus tessellation is different in different races. In addition to different progression rates, the grade of fundus tessellation progression also varies. The progressive lesions in our study were mainly diffuse chorioretinal atrophy, while in Kengo Hayashi et al.’s study, except for progression to diffuse chorioretinal atrophy, lacquer cracks and myopic CNV also appeared. The possible reason was that our population had low myopia diopter, short AL, and a low stretching degree of retinal and choroidal capillaries in the fundus, thus less prone to another myopic maculopathy.

Multivariate logistic regression analysis showed that participants with β-PPA at baseline were more likely to develop new fundus tessellation. The reason may be that β-PPA and fundus tessellation have some common characteristics in formation. In the fundus photograph, β-PPA was characterized by whitish color, visible large choroidal vessels, and visible sclera [23, 24]. In optical coherence tomography (OCT) and tissue sections, β-PPA was characterized by presence of Bruch’s membrane and disappearance of retinal pigment epithelium and choroidal capillary layer [23–25]. The characteristics of β-PPA that the choroidal capillary layer is lost and the visibility of the choroidal large vessel layer increases were similar to that of fundus tessellation, which is characterized by the permeability of the choroidal large vessel. When β-PPA appeared, it indicated that the thinning of the choroidal capillary layer had begun to appear around the optic disc. It also indicated that the thinning of the choroidal capillary layer appeared in other fundus regions, namely, the appearance of the fundus tessellation. The Beijing Eye study [6] showed that there was a significant correlation between fundus tessellation and long AL. However, in our study, we did not find the relationship between them. The probable cause is that the Beijing Eye Study is a population-based cross-sectional study, while our study is a population-based longitudinal epidemiological study. Further studies are needed to confirm whether AL is a risk factor for the development of fundus tessellation.

The Handan Eye Study provides the prevalence and incidence of fundus tessellation in a population-based study and an important theoretical basis for understanding the prevalence of primary myopic maculopathy in a Chinese rural adult population. In addition, this study analyzed the risk factors for the occurrence of fundus tessellation. For people with risk factors, we can closely follow up to find possible myopic fundus changes as early as possible and take effective intervention measures to avoid visual impairment. However, our study also has some limitations: first, the lost to follow-up or excluded population were much older, so the analysis of the effect of age on the appearance of fundus tessellation may be biased. Second, previous studies have shown that choroidal thickness is significantly correlated with fundus tessellation; however, our study did not include the analysis of choroidal thickness. In future studies, we need to further explore the correlation between the fundus tessellation and the choroid. Thirdly, we used qualitative methods to grade the fundus tessellation, which was highly subjective, but the reading results were finally verified and confirmed by a retinal specialist (K.O.M.), so the results were credible.

In conclusion, our population-based longitudinal eye study showed that the prevalence of fundus tessellation was 9.89% and the incidence was 1.21% within 6 years in rural adults in northern China. The 6-year progression rate of fundus tessellation was 1.5%. People with older age and β-PPA were more likely to develop fundus tessellation, which suggests that we need to strengthen the follow-up of these people in clinical work in order to early detect possible fundus lesions and take active intervention measures to avoid visual impairment.

## Data Availability

All data generated or analyzed during this study are included in this article. Further inquiries can be directed to the corresponding author.
